# Role of NFAT in Chronic Lymphocytic Leukemia and Other B-Cell Malignancies

**DOI:** 10.3389/fonc.2021.651057

**Published:** 2021-04-01

**Authors:** Ilenia Sana, Maria Elena Mantione, Piera Angelillo, Marta Muzio

**Affiliations:** ^1^ Division of Experimental Oncology, San Raffaele Hospital IRCCS, Milano, Italy; ^2^ Lymphoma Unit, Department of Onco-Hematology, IRCCS San Raffaele Scientific Institute, Milan, Italy

**Keywords:** nuclear factor of activated T cells, B-cell receptor, chronic lymphocytic leukemia, lymphoma, lymphoid malignancies

## Abstract

In recent years significant progress has been made in the clinical management of chronic lymphocytic leukemia (CLL) as well as other B-cell malignancies; targeting proximal B-cell receptor signaling molecules such as Bruton Tyrosine Kinase (BTK) and Phosphoinositide 3-kinase (PI3Kδ) has emerged as a successful treatment strategy. Unfortunately, a proportion of patients are still not cured with available therapeutic options, thus efforts devoted to studying and identifying new potential druggable targets are warranted. B-cell receptor stimulation triggers a complex cascade of signaling events that eventually drives the activation of downstream transcription factors including Nuclear Factor of Activated T cells (NFAT). In this review, we summarize the literature on the expression and function of NFAT family members in CLL where NFAT is not only overexpressed but also constitutively activated; NFAT controls B-cell anergy and targeting this molecule using specific inhibitors impacts on CLL cell viability. Next, we extend our analysis on other mature B-cell lymphomas where a distinct pattern of expression and activation of NFAT is reported. We discuss the therapeutic potential of strategies aimed at targeting NFAT in B-cell malignancies not overlooking the fact that NFAT may play additional roles regulating the inflammatory microenvironment.

## Introduction

### Targeting B-Cell Receptor Signaling in Chronic Lymphocytic Leukemia

In recent years there have been significant improvements in the field of chronic lymphocytic leukemia (CLL) from both bench and bedside perspectives. CLL cells are addicted to different microenvironmental stimuli with a key role being played by the B-cell receptor (BCR) stimulation and/or constitutive cell autonomous BCR activation (1) leading to cell survival and proliferation. On this scientific basis, several lines of research led to the development of small molecule inhibitors of the kinases that transmit the signals from the proximal BCR signaling complex to the downstream Transcription Factors (TFs) [i.e. Bruton Tyrosine Kinase (BTK) and Phosphoinositide 3-kinases delta (PI3Kδ)] (2, 3). CLL is a disease of the elderly, but it can also affect younger patients, who often are the most difficult to treat given the long disease history and the frequent need for multiple lines of treatment. To date, the only curative approach for CLL patients, unfortunately, is allogeneic bone marrow transplantation, a procedure burdened by a high rate of morbidity and mortality especially in older patients. Nowadays, one of the most powerful available tools for the treatment of CLL, is a novel small molecule that inhibits BTK, Ibrutinib. The drug was approved by the US Food and Drug Administration in 2014 for the treatment of relapsed refractory CLL and for CLL patients with the 17p deletion/mutation, known to frequently be chemo refractory. Ibrutinib was granted approval for first-line treatment of CLL in March 2016; since then, data from real world practice consistently shows a significant improvement in survival curves, in keeping with what investigators previously observed in clinical trials (4, 5). BTK is not the only target of novel non chemotherapeutic agents, as both BTK and PI3Kδ inhibitors are highly effective even for the treatment of refractory or relapsed disease. These drugs are designed to be administered until relapse/progression or unacceptable toxicity. Patients are therefore kept under follow-up during the administration for early detection of signs of clinical progression or toxicity. Several factors have been proposed as predictive markers for the emergence of resistance (i.e. prolonged lymphocytosis) (6, 7), which is currently an unmet clinical need. Novel therapeutic strategies are needed to cure refractory patients and to perhaps achieve deeper response with the intent of fully eradicating the disease.

In this context, we hypothesize that exploring other downstream signaling mediators including transcription factors may reveal novel vulnerabilities of malignant B cells, which could be of aid in treating CLL and other B cell malignancies. Given that resistance to targeted agents often occurs by mutation of the target kinase (8, 9), it is reasonable to hypothesize that blocking downstream signaling molecules could be a strategy to block the transmission of the survival signal to the nucleus. One of the transcription factors involved in B-cell antigen receptor signaling is NFAT, Nuclear factor of activated T-lymphocytes [others being Nuclear Factor kappa-light-chain-enhancer of activated B cells (NFκB), cMyc and activator protein 1 (AP1)]. Here, we briefly describe the biology of NFAT followed by a discussion on the expression pattern and functional role of NFAT transcription factors in CLL and other lymphoid malignancies.

### The Nuclear Factor of Activated T-Cells Family of Transcription Factors

**Figure 1:**
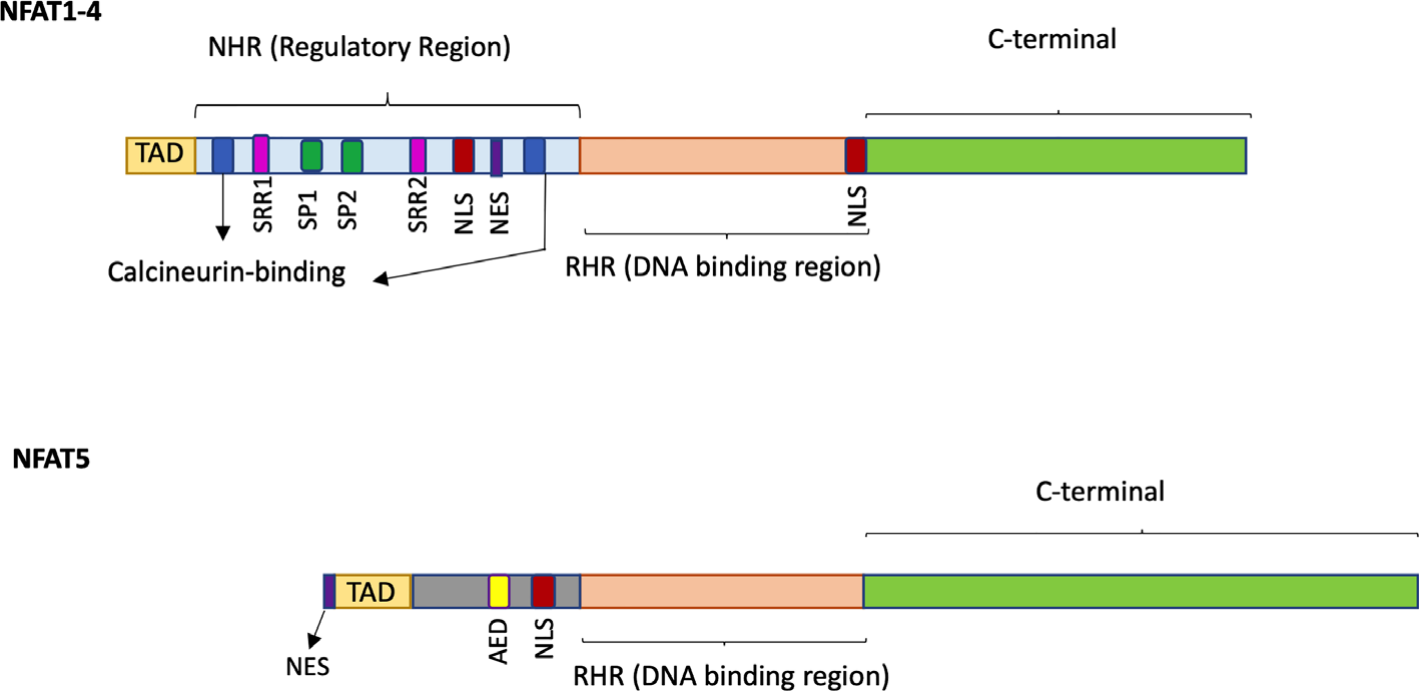
Schematic representation of the structure of NFAT1-4 and NFAT5. NFAT proteins contain a REL homology region (RHR), the most conserved domain, which binds to DNA and is common to all five NFAT family members. The NFAT homology region (NHR) is the regulatory region conserved in NFAT1-4 members but not in NFAT5 and contains two calcineurin-binding sites, serine residues (SSR, SP), a Nuclear Localization Sequence (NLS) and a Nuclear Export Signal (NES). NFAT5 does not display the calcineurin binding site but owns an auxiliary export domain (AED) and a NLS and possess a nuclear export sequence (NES) located at the first 19 amino acids. All NFAT family members have a N-terminal Transactivation Domain (TAD) and a C-terminal domain which are the less conserved regions.

The NFAT family of TFs includes five members grouped by the presence of the REL homology region (RHR), a highly conserved DNA binding domain that confers a unique DNA binding specificity to these proteins (see **Figure 1** for a schematic representation). Several alternative names exist for each NFAT member, and they are all reported in **Table 1** together with essential information on each gene. Herein, we refer to the NFAT family members with their official names; NFAT1, NFAT2, NFAT3, NFAT4, and NFAT5.

**Table 1:** Summary of key features of human NFAT genes, mRNAs and proteins.

Name	Aliases	Chromosome	n° of splice variants (Uniprot)	n° of splice variants (Ensembl)	total n° of isoforms	n° of different protein coding variants	UniprotKB entry	Ensembl entry	NCBI entry	RNA expression in tissues*	RNA expression in blood*
NFAT1	NFATc2, NFATp, NF-ATP	20q13.2	5	7	7	6	Q13469	ENSG00000101096	4773	Appendix, Lymph node, Tonsil	γδT cell, NK- cell, memory CD8 T-cell
NFAT2	NFATc1, NFATc, Nf-ATC, NF-ATc1.2	18q23	10 (+3)	18	19	11	O95644	ENSG00000131196	4772	Appendix, Tonsil, Lymph node	Non classical monocyte, Naive B cell, intermediate monocyte, memory B cell
NFAT3	NFATC4, NF-ATc4	14q12	24 (+3)	33	34	22	Q14934	ENSG00000100968	4776	Ovary, Cervix uterine, urinary bladder, gall bladder	not detected
NFAT4	NFATC3, NFATX	16q22.1	6 (+6)	22	25	5	Q12968	ENSG00000072736	4775	Thymus	γδT cell, memory CD8 T-cell, ^MAIT T cell, Naive CD8, NK-cells
NFAT5	TONEBP, NFATL1, KIAA0827, OREBP, NFATZ, NF-AT5	16q22.1	5 (+6)	13	14	4	O94916	ENSG00000102908	10725	Parathyroid gland, vagina, placenta, skin, bone marrow	Neutrophil, non classical monocyte

*Tissues and cell types with relative high levels of mRNA expression are listed based on consensus data from Human protein atlas.

^MAIT T cell indicates Mucosal associated invariant T cell.

**Figure 2:**
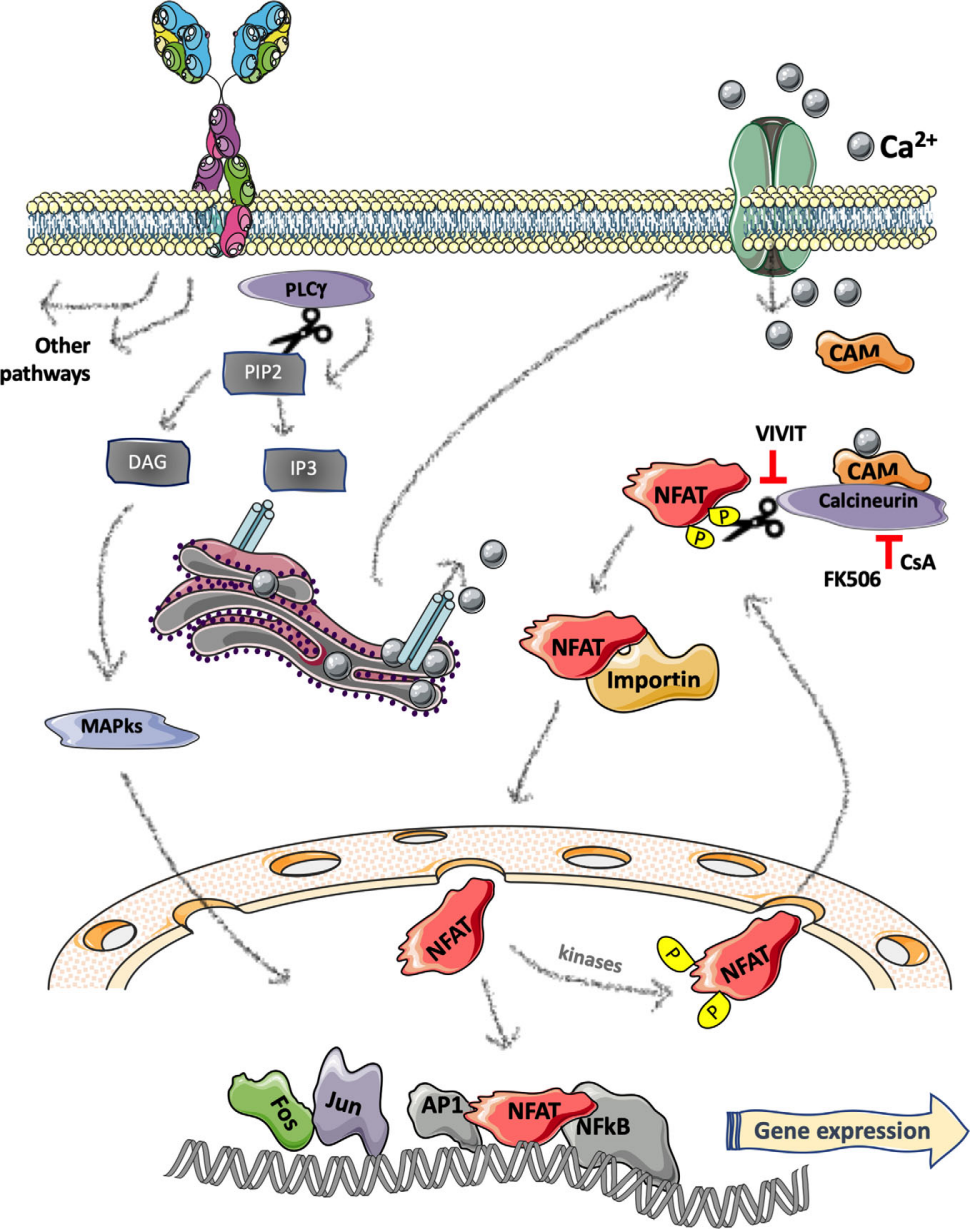
Schematic representation of B-cell receptor induced NFAT pathway. Stimulation of the B-cell receptor (BCR) activates a cascade resulting in phospholipase C (PLCγ) activation and hydrolyzation of phosphatidylinositol-3,4-bisphosphate (PIP_2_) generating the release of two second messengers: diacylglycerol (DAG) and inositol-1,4,5-trisphosphate (IP3). IP3 binds to the IP3 receptor located on the endoplasmic reticulum leading to release of Ca^2+^ from the ER and an extracellular influx of Ca^2+^ in the cytosol that causes the binding of calmodulin (CaM) to the calcineurin phosphatase and the consequent dephosphorylation and activation of NFAT. The nuclear translocation of NFAT is mediated by nuclear importing factors (e.g., importin). In the nucleus, NFAT binds to the DNA either alone or with other factors regulating gene expression. **Figure 2** was created using Servier Medical Art (https://smart.servier.com).

Four NFAT proteins share sequence homology in the N-term regulatory region (NHR) responsible for the modulation by Calcium signaling, while NFAT5 is induced by osmotic stress (10). Briefly, calcium-dependent NFATs are normally retained in an inactive state into the cytoplasm of the cells by different kinases that phosphorylate the NHR domain (11). The stimulation of receptors such as the BCR in B-cells and the T-cell receptor (TCR) in T-cells (12) generates a cascade that induces calcium mobilization through the activation of phospholipase C (PLCγ) that hydrolyzes phosphatidylinositol-3,4-bisphosphate (PIP_2_) leading to the release of diacylglycerol (DAG) and inositol-1,4,5-trisphosphate (IP3). IP3 binds to the IP3 receptor and causes the release of Ca^2+^ from the endoplasmic reticulum and the consequent extracellular influx of Ca^2+^ in the cytosol by specific calcium channels (13, 14) (see **Figure 2** for a schematic representation). The presence of Ca^2+^ ions cause the binding of calmodulin (CaM) to the calcineurin phosphatase, leading to the dephosphorylation and activation of NFAT serine residues in the regulatory domain. Nuclear importing factors then mediate NFAT translocation into the nucleus where it binds the DNA alone or with other factors such as AP1, Stat3, GATA, c-Fos, c-Jun, and NFκB, to regulate gene expression either activating or silencing target genes; most of which are immune-related (15, 16). NFAT inactivation and the following relocation into the cytoplasm is operated by several kinases including glycogen-synthase kinase 3β [please refer to the following recent reviews for molecular details on NFAT proteins (16–19)].

The molecular activation of NFAT5 is more complex and still partially defined. Under isotonic conditions, there is a continuous shuttling of NFAT5 between the cytoplasm and the nucleus that can be regulated by tonicity stress. While hypotonic stress promotes nuclear export of the protein, hypertonic conditions induce transcription, translation and nuclear import of NFAT5 (20, 21). With its well-known tonicity-related regulatory role, it is emerging that several isotonic stimuli can promote NFAT5 activity; for instance, triggering of innate immunity receptors such as Toll-like receptors and consequent activation of reactive oxygen species (ROS) and mitogen activated protein kinases (MAPK) results in NFAT5 activation that, interestingly, shows distinctive features with respect to the osmotic activated response (22). However, the interplay between these two mechanisms of activation and the exact molecular pathways involved are still elusive and yet to be fully uncovered.

### Studies on Different NFAT Deficient Mice Uncover Distinct Functional Roles in the B-Cell Lineage

To dissect the role of these TFs, several studies analyzed the phenotype of specific NFAT-deficient mice as well as combinations of two or more genetic deletions. For a comprehensive description of the murine lines please refer to online resources (e.g., the International Mouse Phenotyping Consortium: https://www.mousephenotype.org; MGI-Mouse Genome Informatics-: http://www.informatics.jax.org/). Herein, we focus on the results related to lymphocytes and the B-cell lineage as this may help in understanding and interpreting the specific role of individual NFAT proteins in B-cell malignancies.

With the notable exception of NFAT3, for which no specific studies analyzed the B-cell compartment, deletion of individual NFAT or double inactivation of NFAT genes *in vivo* revealed the importance of this TF family in the regulation of lymphocyte differentiation, proliferation, apoptosis, cytokine production, and inflammation. The vast majority of the immunological studies, as well as the B cell-centered ones, focused on NFAT1 and NFAT2 that regulate different B-cell populations in diverse ways (23). Between the two, NFAT2 deficiency has a more severe impact; indeed, the global loss of NFAT2 determines prenatal lethality around day 14/15 upon gestation (24, 25). However, chimeric mice with lymphocyte-restrained NFAT2 loss showed defects in BCR-mediated proliferation of B cells (26), and a peculiar deficiency in peritoneal CD5+ B1a B-cells (27). Specifically, the defect in BCR-induced proliferation is determined by NFAT2-dependent expression of CD22, Rcan1, Tnfsf14, FasL and other key proteins of the BCR signaling pathway. In addition, the abrogation of NFAT2-mediated calcium flux response facilitates activation-induced cell death (AICD), which leads to the loss of CD5+ peritoneal B1a cells. Moreover, the lack of NFAT2-mediated repression of IL-10 production, impacts on IFN-γ production by CD4+ T-cells, impairing the capacity of B-cells to stimulate T cell proliferation (28). Mice lacking NFAT2 in pro-B cells have deficient expression of an essential TF determining B-cell lineage fate, EBF1, and a similar phenotype of EBF1-deficient mice (29) with defective Immunoglobulin (Ig) gene rearrangement, and pre-BCR formation which impairs B cell development and leads to severe B-cell lymphopenia (30). The early developmental role of NFAT2 in peripheral B-cells was endorsed by a recent study that characterized lymphocyte dissemination in mice bearing deletion of NFAT2 in CD19-positive cells. The authors confirmed NFAT2-dependent deficiency in peritoneal CD5+ B1a B-cells that was accompanied by increased immature and mature follicular B cell populations (31). At later stages, NFAT2 loss of function causes functional defects only of mature B cells that promote mild clinical course of experimental autoimmune encephalomyelitis (28).

NFAT1-null mice have a normal development and a less severe phenotype. Nevertheless, after 6 months a proportion of the litters showed alterations of the immune system such as lymph node hyperplasia and splenomegaly accompanied by enlarged germinal centers and pronounced retardation in the involution of the thymus. NFAT1 deficient mice also displayed a hyperproliferative syndrome, higher B and T cell counts, dysregulated production of IL-4, and higher primary and secondary immune responses (32–34). Notably, NFAT1 plays a pivotal role in regulating the response of B-cells to self-antigen, balancing the processes of anergy and self-tolerance (35). Moreover, NFAT1 in B cells, controls and represses the expression of Cyclin E1 and E2, taking control of cell cycle progression and proliferation rates (36).

NFAT4 shows a more restricted role in regulating T cell activity; however, double deficient mice for NFAT1 and NFAT4 experience lymphadenopathy, splenomegaly, and a strongly activated phenotype with a substantial increase in serum IgE and IgG1 levels, similar to single knock-out mice (37). One of the origins of the lymphadenopathy was attributed to the observed resistance to apoptosis, due to decreased FasL expression and defective AICD induction (38). Moreover, the absence of both NFAT1 and NFAT4 drives naive CD4 T cells into Th2 cell differentiation even in the absence of endogenous IL-4, and boosts their responsiveness to TCR-mediated activation and secretion of Th2-type lymphokines (39). The elevated Th2 cytokine production also leads to hyperactivation of mature follicular B cells but not of marginal zone (MZ) B cells. This evidence indirectly links the loss of both NFAT1 and NFAT4 to the altered B cell phenotype of these mice, which have a lower representation of MZ B cells and a higher number of mature follicular B cells (40).

Focusing on NFAT5, the tonicity-responsive member of the family, its complete loss of function results in gestational lethality. Heterozygous animals show a phenotype marked by lymphoid hypocellularity, with thymus and spleen hypoplasia, defective antigen-specific antibody responses (in particular IgG secretion) and less mature CD4 and CD8 cells in the spleen and lymph nodes (41). These indications highlight the role for NFAT5-mediated adaptation to physiologic osmotic stress for lymphocyte-mediated immunity, with a putative B-cell centered role on T-cell dependent Ig response and proliferation, specifically under hypertonic conditions (42). Interestingly, NFAT5 showed a tonicity-independent role in the development and activation of macrophages where NFAT5 accumulation and the following increased expression of target genes such as TNF and IL6 can be mediated by Toll-like receptors and NFκB pathway activation (43). NFκB-mediated expression of NFAT5 also has a crucial role in pre–T-cell receptor thymocytes where it regulates the expression of the prosurvival factors A1 and Bcl2, and attenuates the proapoptotic p53/Noxa axis (44).

From all these observations it emerges that not only are NFAT1, NFAT2, and NFAT4 involved in the regulation and homeostasis of B-cells and BCR signaling, but NFAT5 also plays a crucial role which has yet to be fully characterized. On the contrary, no functional data are available for NFAT3. Nonetheless, both NFAT1 and NFAT2 are expressed by distinct B-cell malignancies as described in detail below, while less information is available on NFAT3, NFAT4, and NFAT5.

## NFAT Expression and Activation in CLL and Other Lymphoid Malignancies

### Expression and Function of NFAT in B-Cell Lymphomas

The category of non-Hodgkin lymphomas (NHL) comprises a large spectrum of entities ranging from indolent to highly aggressive diseases (45). Pathogenesis of most NHLs is unknown. For some subtypes, a chronic immune stimulation role has been suggested, thus, gaining insight on the multistep mechanism that leads to malignant transformation is key for the development of new treatments.

NFAT has neither been highlighted as a prominent B-cell lymphoma-associated molecule, nor a frequently mutated gene; however, distinct studies reported specific molecular and functional features of NFAT family members that may open up interesting novel therapeutic perspectives.

First, NFAT2 could be detected by IHC in lymphoid cells in routine biopsies of several hematologic malignancies, while nuclear NFAT2 was observed in a proportion of Burkitt and diffuse large B cell lymphoma (DLBCL) samples, suggesting an ongoing activation of the pathway in this type of lymphoma (46). In contrast, NFAT2, but not NFAT1, is downregulated by promoter methylation in Hodgkin’s lymphoma cells (47). To note, the pattern of expression of NFAT does not fully reflect its role, as the activity of this TF is regulated by different mechanisms that eventually control the shuttling between the nucleus and the cytoplasm.

By using patients’ derived cells and cell lines, Pham et al. not only reported constitutive activation of NFAT2 in large B-cell lymphoma, but also demonstrated that NFAT2 and NFκB cooperate to drive CD40L expression, which in turn triggers pro-survival signaling. NFAT siRNA inhibitors as well as drugs targeting NFAT activation blocked CD40L expression and induced apoptosis (48). The same group also demonstrated that both large B-cell lymphoma and mantle cell lymphoma constitutively express NFAT1 and NFAT2 that control BLyS expression and survival signaling again in cooperation with NFκB (49). Another interesting target of NFAT2 is c-Myc that can be transcriptionally upregulated by NFAT2 through an epigenetic chromatin remodeling in DLBCL (50).

On the contrary, a tumor suppressive role of NFAT1 was proposed in DLBCL where its downregulation correlated with increased cyclin E expression; specifically, NFAT1 directly controlled cyclin E induction by binding to its promoter in lymphoma cell lines (36). Moreover, NFAT1 was implicated in mediating BCR-induced cell death in Hodgkin lymphoma cells, a phenomenon observed in selected cell lines; both Cyclosporin and FK506 inhibited apoptotic signaling, further supporting the functional involvement of NFAT (51).

Regarding the mechanisms responsible for NFAT overexpression and activation in lymphoma patients, while most of the studies suggest transcriptional and/or post-transcriptional mechanisms of regulation of NFAT in B-cell lymphoma, there is also evidence of NFAT gene amplification in a proportion of DLBCL of the ABC type (52). An increase of hexosamine biosynthetic pathway and O-GlcNAc metabolism plays a critical role in DLBCL cell proliferation, and is responsible for the observed NFκB and NFAT activation (53). Constitutive ongoing BCR signaling may also explain constitutive NFAT2 activation in DLBCL (54). However, a recent report suggested a BCR-independent, Calcium-dependent pathway towards NFAT2 activation in DLBCL (55, 56).

Finally, in addition to protein phosphorylation and splicing, distinct post-translational modifications modulate its activity including acetylation, SUMOylation and cleavage (57). However, no specific data on these mechanisms operating in B-cell malignancies are available.

### Expression Pattern and Functional Role of NFAT in CLL

CLL involves mature clonal B-cells that accumulate in the peripheral blood and lymphoid organs where they receive supportive signals form the microenvironment. The BCR, the key protein for every B-lymphocyte, not surprisingly modulates CLL cells biology as well; several signaling molecules downstream of the BCR such as kinases and TFs are involved, and they have been recently targeted for therapeutic purposes (58, 59). Among the most relevant BCR-mediated TFs, we focus on NFAT, not overlooking the fact that NFκB as well as other TFs play a relevant role and may complement the activity of NFAT itself.

In 1996, Schuh et al. demonstrated for the first time that in contrast to normal B-cell, malignant cells isolated from the peripheral blood of patients with CLL show nuclear/active NFAT1 even in the absence of *in vitro* stimulation; in parallel, NFκB and AP1 activation were also observed (60). CLL cells show higher mRNA levels of expression of both NFAT1 and NFAT2 as compared to normal lymphocytes (61); hypomethylation of the NFAT2 promoter region as well the first intron region may explain higher levels of both mRNA and protein in CLL as compared to normal B-cells types (62). A comprehensive study on the epigenome and regulatory chromatic landscape of CLL highlighted that active chromatin regions were enriched for binding motifs of NFAT (as well as FOX and TCF/LEF transcription families) (63). When different groups of CLL patients with different clinic-biological characteristics were analyzed, NFAT promoter hypomethylation correlated with clinical staging (64). More recently, looking for markers of a specific subset of CLL, bearing trisomy of chromosome 12, Abruzzo at el. discovered that NFAT1, NFAT2, and NFAT4 mRNAs are significantly overexpressed (65).

Based on all these somewhat descriptive analyses, several authors suggested that BCR-mediated NFAT2 overexpression may be implicated in CLL pathobiology and may potentially be targeted for therapeutic purposes. Along this line of reasoning, several questions emerged: Which are the target genes of NFAT transcription factors in leukemic cells? Which are the functional consequences of NFAT hyperactivation in CLL? Which are the molecular mechanisms regulating NFAT activation in malignant cells?

No specific NFAT ChIP-seq analysis was performed in CLL cells, thus hampering a broad view of all its target genes; nevertheless, independent studies demonstrated that distinct genes that are typically expressed by leukemic cells are directly regulated by NFAT family members in different cell types.

CD23 is a receptor for FcE, and a distinctive molecule expressed on the surface of CLL cells as well as released in the serum (66). Two different isoforms regulated by two different promoters exist, namely CD23a and CD23b. The CD23b promoter is specifically regulated by NFAT1 and NFAT2 in concert with STAT6 (67); in contrast, CD23a expression is regulated by Notch2 (68). CD23 expression can also be upregulated by BCR stimulation in CLL cells where blocking NFAT prevents CD23 induction (61).

CD5 expression is regulated by NFAT in normal B-cell populations (27, 69, 70); however, it is not known if the same occurs in CLL where it is distinctively expressed on the cell surface.

LCK was recently identified as a direct NFAT2 target gene in both human and mouse CLL samples (71). CD5 mediated IL10 production is regulated by NFAT in CLL cells; by cooperating with STAT3, NFAT2 binds to IL5 and IL13 promoters and the IL10 enhancer to upregulate their expression (72).

NFAT2 is constitutively active in approximately half of CLL cases, the same than that characterized by concomitant MAPK phosphorylation and anergy in terms of response to the BCR. Despite constitutive basal levels of NFAT2 activation, it can be further induced after BCR stimulation, at least in a group of responding cases that are characterized by an adverse clinical outcome (61). Not only is NFAT overexpressed and activated in CLL, but NFAT binding sites are hypomethylated in leukemic samples suggesting the overactivation of target genes that may be related to autoreactive BCR (73).

To assess the functional role of NFAT during disease development and/progression, different approaches were used including ablation of NFAT2 in a mouse model of leukemia, and the use of drugs targeting NFAT activation. The two approaches address different questions whilst also being complementary; while genetic inactivation results in complete inhibition of a single molecule *in vivo* over time, drug treatment suddenly interrupts different NFAT family members as well as any additional off targets. Here, we focus on genetic approaches while describing potential drug targeting in a dedicated paragraph below for both CLL and lymphoma.

Overexpression of the TCL1 oncogene in the B-cell lineage results in the development of a malignant disease resembling human CLL, as characterized by the accumulation of CD5-positive clonal B-cells in the peripheral blood and lymphoid organs (74); this is a widely used and accepted mouse model of leukemia. Leukemic cells of this model show constitutive activation of NFAT2 and somewhat anergic features (71), as previously shown for a group of CLL cases (75). Genetic inactivation of NFAT2 in the B-cell types of these mice led to rapid acceleration of leukemia development and progression toward and aggressive disease resembling Richter transformation, occurring in a small proportion of CLL patients (71). The authors suggest that NFAT2 is a key regulator of anergy in CLL. To better understand if NFAT deletion impacted directly on different BCR downstream signaling or rather on clonal selection of different BCR recombination, Muller et al. analyzed clonal evolution in leukemic mice and found that NFAT2 signaling in CLL cells precipitates the oligoclonal selection of preferentially unmutated BCRs (76).

Overall, these data suggest that NFAT2, being implicated in the maintenance of anergy, may restrict leukemia development; however, at the same time, anergic signaling provides a survival advantage to the cells. It is important to evaluate the effect of NFAT2 inhibition after leukemia development, as data from primary patient samples suggest that it may have therapeutic activity by interrupting anergy (77).

### Therapeutic Perspectives

Given the central role of NFAT in regulating the adaptive immune response, it has been thoroughly scrutinized as a “specific” drug target to achieve immunosuppression in the context of organ transplantation, or to dampen excessive autoimmune manifestations. The most widely used drugs targeting the NFAT pathways are Cyclosporine A (CsA) and Tacrolimus, both originally isolated from fungi, acting with slightly different mechanisms of action. Briefly, as schematically reported in **Figure 2**, they inhibit the activity of the phosphatase Calcineurin, thus augmenting the phosphorylation state of all its substrates including NFAT1-4 family members; to do so, CsA binds to Cyclophilin while FK-506 (an alternative name of Tacrolimus) binds to FKBP12 (78). Both drugs are widely used to prevent graft-rejection or to treat autoimmune diseases; however, several years ago, anecdotical reports suggested its potential positive effect in the context of CLL (79, 80).

Nowadays, accumulating preclinical data from many different labs suggest that targeting NFAT may represent a novel therapeutic approach to treat at least a subset of NFAT-positive B-cell malignancies. In particular, CsA, FK-506 as well as a short cell-permeable NFAT-specific inhibitory peptide (called VIVIT as according to its core aminoacidic sequence) have been tested *in vitro* and *in vivo* in mouse models of leukemia and lymphoma (81, 82). To note, VIVIT peptide directly binds to the NFAT-docking portion of Calcineurin, thus being more selective towards these transcription factors as compared to CsA.

Targeting CLL cells with the VIVIT peptide blocks BCR-mediated NFAT2 activation and target genes in responsive cells (61, 71); at the same time, VIVIT blocks constitutive NFAT activation in CLL, thus rescuing leukemic cells from anergy (77). More importantly, targeting spontaneous NFAT activation with VIVIT not only blocked related biochemical pathways but also induced cell death *in vitro* and delayed leukemia progression *in vivo* in mouse models (77). These observations suggest that NFAT controls survival signals in anergic cells, though, at the same time keeps the cells in an indolent state. In fact, as mentioned above, deleting the whole NFAT2 gene from cells before leukemia development, triggers faster accumulation of the disease and progression (71).

Cyclosporin and FK506 inhibited NFAT signaling leading to the abrogation of pro-survival effects present in CLL cells, even in the presence of supportive signaling given by stroma cells (64). Interestingly, the use of the BTK signaling inhibitor, a clinically approved drug targeting upstream BCR signaling, abrogated NFAT activity in leukemic cells suggesting that downstream NFAT activation may represent a novel therapeutic target in the cases where resistance to Ibrutinib arise due to mutations in upstream molecules of the signaling cascade (64).

Targeting the upstream BTK kinase with different inhibitors, including Ibrutinib, blocks NFAT2 together with its targets including IL10; to note, IL10 can control PDL1 expression preferentially in the ABC subset of DLBCL thus suggesting that targeting NFAT may impact on the expression of this molecule, therefore being relevant for anti-tumor immunity (54). Along this line, a recent paper confirmed chronic NFAT activation in ABC-DLBCL controlling IL10 production and demonstrated the efficacy of calcineurin inhibitors in blocking NFAT signaling and reducing proliferation. However, the authors proposed a BCR-independent mechanism of NFAT activation, yet a BCR-dependent expression of NFAT protein that is dependent on NFκB signaling. Interestingly, blocking calcineurin synergized with BCL2 and MCL1 inhibitors to kill lymphoma cells (55).

Based on all these observations, using CsA, FK-506, or novel formulations of VIVT appear to be a promising therapeutic perspective. However, the concomitant immunosuppressive activity of these drugs may potentially counterbalance the direct anti-tumor activity, and it should be carefully considered for any putative future clinical approach (83). Along this line, it will be crucial to design and engineer novel drugs targeting NFAT in the malignant cells only. Finally, several additional drugs targeting NFAT are emerging, and it will be important to test them either alone or in combination with signaling inhibitors using advanced preclinical models and primary tumor samples.

## Discussion

The B-cell receptor is the key molecule regulating the pathobiology of both normal and malignant B-lymphocytes; accordingly, targeting the kinases proximal to the BCR (such as BTK and PI3Kδ), has emerged as a successful treatment strategy. Nevertheless, BCR targeting is in some cases not sufficient to achieve disease control and, eventually, tumor cells find alternative mechanisms to survive and proliferate. For this reason, efforts devoted to studying and identifying additional new potential druggable targets along the BCR signaling cascade are warranted. In this review, we explored the biology of a key family of transcription factors that are activated after BCR stimulation, namely NFATs.

First, to obtain insight into the intrinsic role of different NFATs in the B-cell context, we reported an overview of genetically modified mice where a dual role of NFAT1 and NFAT2 emerged, regulating both cell proliferation and cell death after BCR stimulation. Next, we detailed the expression, activation status, and functional role of different NFAT family members in CLL and other B-cell malignancies. NFAT1 and NFAT2 were described to be not only overexpressed but also functionally implicated in the regulation of malignant B-cell biology. In contrast, no information was available on NFAT3 and NFAT4 in this context, while a recent paper demonstrated that NFAT5 is overexpressed in CLL where it facilitates malignant cells survival and activation (84); yet, NFAT5 activation is not dependent on BCR but it is regulated by osmotic stress and inflammatory stimuli. Overall, we described a cell autonomous function of BCR-related NFAT activation in leukemia and lymphoma cells. Targeting this molecule using a specific inhibitor was shown to be beneficial in treating CLL and lymphoma in preclinical models; however, NFAT deletion in mouse models broke anergy with a paradoxical induction of leukemia progression *in vivo* (85).

It has been known for years now that the NFAT family of transcription factors are involved in several processes which are central for immune system function, inflammation, and the development of both autoimmune and neoplastic diseases (86). This is particularly important in light of the role that the tumor microenvironment has been recognized to have for tumor survival and progression. NFAT may exert additional effects in different cell types, including stroma cells, by regulating inflammation and inflammation-associated cancer, as previously reported by other reviews on this topic (86, 87). To note, inflammation is a hallmark of CLL as well as other lymphoid malignancies where infiltrating immune cells, stroma, and vessels contribute to shape a complex tumor microenvironment (88–90). With this in mind it is reasonable to hypothesize that inhibition of the NFAT pathway could be effective for the treatment of lymphoproliferative disease since it affects cell function and survival both on and off the tumor. Recently Bucher et al. have reported strong evidence showing that NFAT signaling is chronically activated in DLBCL regulating cell survival and inflammatory cytokines (55, 56). In particular ABC DLBCL cells seem to be particularly dependent on the activation of the NFAT pathway. Moreover, data shows that blockade of signals generated from BCR activation is not able to affect NFAT1/2 phosphorylation or translocation to the nucleus. These findings suggest that other mechanisms could be responsible for the pathway activation. In summary, evidence has accumulated showing that the NFAT family controls biological processes on and off the tumor, which should be carefully analyzed in the context of targeting for any future treatment of lymphoproliferative diseases.

## Author Contributions

All authors listed have made a substantial, direct, and intellectual contribution to the work and approved it for publication.

## Funding

This work was supported by Ministero della Salute under Ricerca Finalizzata 2018–ID. RF-2018-12367072 project—P.I. MM and co-P.I. PA; AIRC under IG 2019–ID. 23088 project—P.I. MM.

## Conflict of Interest

The authors declare that the research was conducted in the absence of any commercial or financial relationships that could be construed as a potential conflict of interest.

## Abbreviations

BCR, B cell receptor; BTK, Bruton Tyrosine Kinase; CLL, Chronic Lymphocytic Leukemia; DLBCL, Diffuse large B-cell lymphoma; NFAT, Nuclear Factor of Activated T cells; NFκB, Nuclear Factor kappa-light-chain-enhancer of activated B cells; PI3K, Phosphoinositide 3-kinases; TF, Transcription Factor.
